# Declines and recovery in endangered Galapagos pinnipeds during the El Niño event

**DOI:** 10.1038/s41598-021-88350-0

**Published:** 2021-04-22

**Authors:** Diego Páez-Rosas, Jorge Torres, Eduardo Espinoza, Adrian Marchetti, Harvey Seim, Marjorie Riofrío-Lazo

**Affiliations:** 1 Galapagos Science Center. Isla San Cristóbal, Universidad San Francisco de Quito, Islas Galápagos, Ecuador; 2 Departamento de Ecosistemas Marinos, Dirección Parque Nacional Galápagos, Islas Galápagos, Ecuador; 3 Department of Marine Sciences, The University of North Carolina at Chapel Hill, Chapel Hill, NC USA

**Keywords:** Climate-change ecology, Population dynamics, Marine biology, Zoology, Biodiversity

## Abstract

Currently, the Galapagos sea lion (GSL, *Zalophus wollebaeki*) and Galapagos fur seal (GFS, *Arctocephalus galapagoensis*) are among the most important endemic species for conservation in the Galapagos Archipelago. Both are classified as “Endangered” since their populations have undergone drastic declines over the last several decades. In this study we estimated the abundance of both otariids, and their population trends based using counts conducted between 2014 and 2018 in all their rookeries, and we analyzed the influence of environmental variability on pup production. The GSL population size in 2018 in the archipelago was estimated to be between 17,000 to 24,000 individuals and has increased at an average annual rate of 1% over the last five years after applying correction factors. The highest number of GSL counted in the archipelago was in 2014 followed by a population decline of 23.8% in 2015 that was associated with the El Niño event that occurred during that year. Following this event, the population increased mainly in the northern, central and southeastern rookeries. The GSL pup abundance showed a decreasing trend with the increase in intensity of the El Niño. The GFS population in 2018 was counted in 3,093 individuals and has increased at an annual rate of 3% from 2014 to 2018. A high number of GFS counted in 2014 was followed by a population decrease of 38% in 2015, mainly in the western rookeries. There was interannual population fluctuations and different growth trends among regions of the archipelago. GSL and GFS pup abundance has a strong decreasing tendency with the increase in the subthermocline temperature (ST) and the El Niño 1 + 2 index. Our results provide evidence that both species are highly vulnerable to periodic oceanographic-atmospheric events in the Galapagos Archipelago which impact prey abundance and the flow of energy in the unique Galapagos ecosystem.

## Introduction

Population assessments for marine mammals are challenging but necessary as they provide information for management success^1,2^. Generally, these evaluations are more accurate for species that breed on land or near the coast, such as pinnipeds and sea otters^3,4^. However, the proportion of the population that is ashore can be challenging to determine, since breeding rookeries can be difficult to access and, in some cases, are geographically dispersed^5,6^. Estimating the abundance of otariids presents other challenges, since there are an unknown proportion of adults at sea that are not counted during a count^7,8^. However, pups are confined to land in breeding rookeries for the first few months of life, so their numbers are more constant^6,9^. Therefore, pups can be used as an indicator of abundance and a basis for estimating population trends^10,11^.

Density-dependent (ecological conditions) and independent factors (environmental and anthropogenic conditions) are expected to influence population dynamics of pinnipeds^12,13^. Environmental variability influences the distribution and abundance of otariids^14,15^, as these species use specific areas during their sea foraging trips^16,17^. Oceanographic variability, among other factors, also influences pup production rates^18,19^. For example, environmental perturbations such as El Niño–Southern Oscillation (ENSO) decrease the productivity levels in marine ecosystems reducing the availability of main prey of marine predators^20,21^. This results in nutritional stress and increase in population mortality rates, especially in species that inhabit tropical systems^14,22^.

**Figure 1:**
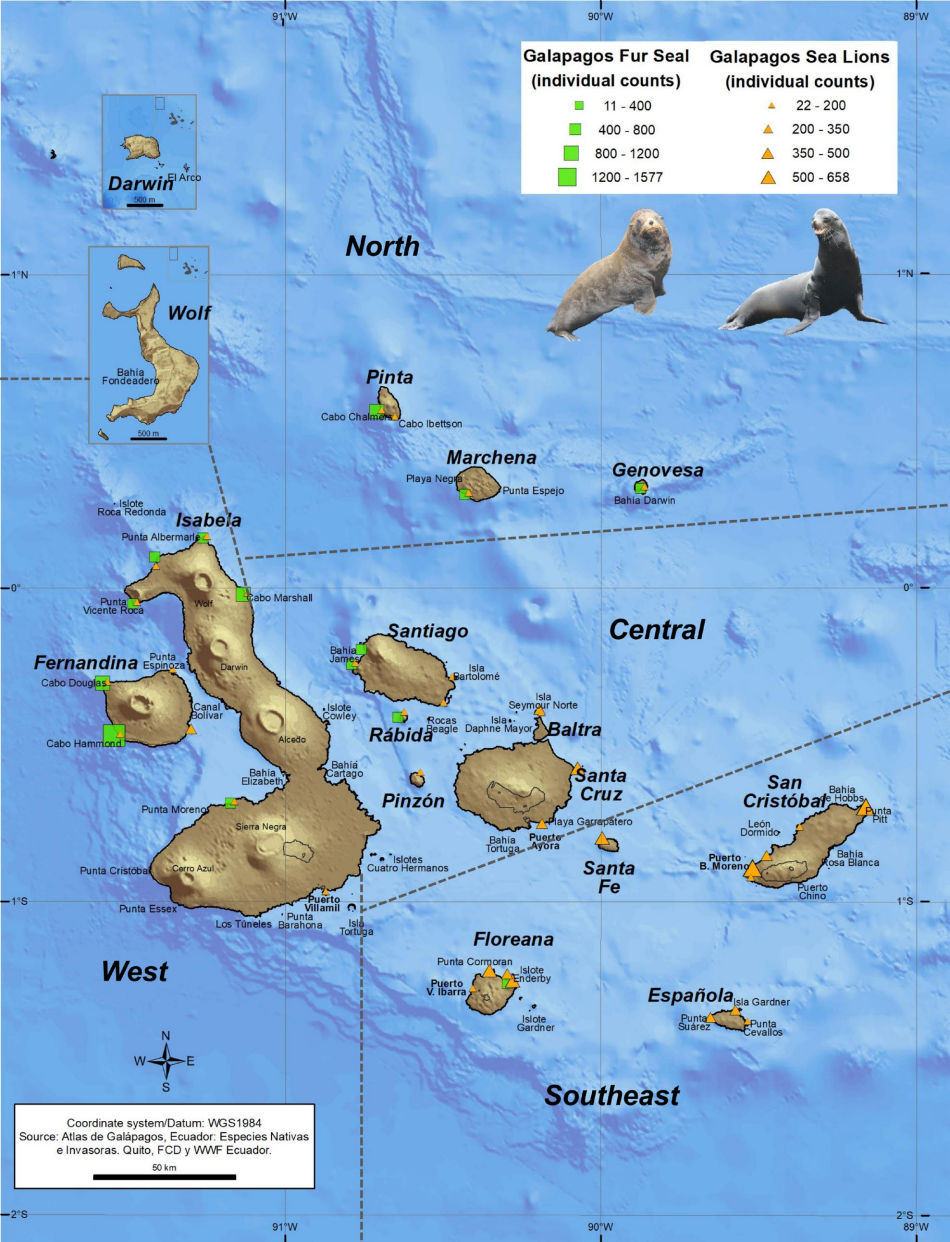
Breeding rookeries of the Galapagos sea lion (orange symbols) throughout the Galapagos Archipelago and Galapagos fur seal (green symbols) in western and northern islands of the archipelago. The symbol sizes are according to the individuals’ abundance. The regionalization scheme of the archipelago is shown. (*Map courtesy of Byron X. Delgado, GIS Research / Knowledge Management, Charles Darwin Foundation*).

The Galapagos sea lion (GSL, *Zalophus wollebaeki*) and Galapagos fur seal (GFS, *Arctocephalus galapagoensis*) are otariids endemic to the Galapagos Archipelago (Fig. 1), adapted to this hotspot of local productivity in the midst of a tropical environment in the equatorial Pacific^23^. This creates an ecological challenge for these species, as they depend on a surrounding ocean with generally poorer conditions than other otariids living at higher latitudes^24,25^. The GSL is the most abundant of the two otariids and it is distributed throughout the archipelago, with larger rookeries on islands of the southeastern region^10^, while the GFS breeds on eight main rookeries in the western and northern islands, with its largest population on Fernandina island^26^. Currently, both species are classified as Endangered in the Red List of the International Union for Conservation of Nature (IUCN) as their populations have undergone a drastic decline in the last few decades^27,28^. Consequently, these otariids are among the species with the highest priority for conservation in the Galapagos Marine Reserve^29^.

There is limited published data to estimate the actual population sizes and growth trends of these species, despite their year-round presence in the archipelago and their high fidelity to their breeding rookeries^10,26,30^. However, performing simultaneous counts is complex due to the large distance between the breeding sites^29^. Overall populations of GSL and GFS were estimated at approximately 16,000 and 8,000 individuals, respectively, according to a census in 2001^26^. Therefore, to date, it is known with certainty that populations have decreased by 50% since the last global census of 1978, when the populations were estimated at 40,000 and 16,000 individuals of GSL and GFS respectively^30^. These population decreases are related to the effects of oceanographic-atmospheric disturbances such as the ENSO, that occur in the Pacific Ocean and affect the Galapagos Archipelago, leading to a lack of food resources in the marine environment^24^. These effects are exacerbated when combined with anthropogenic stressors that contribute to the deterioration of their habitat^10^.

Oceanographic variability in the Galapagos Archipelago causes regional changes in marine productivity that are reflected in the ecological conditions of GSL populations throughout the archipelago^31^. These changes are influenced by the major ocean features that make the western region highly productive through the strong upwelling caused by the Equatorial Undercurrent, the central and southeast regions less productive through localized upwelling, while the north region has the lowest productivity levels due to its proximity to the Equatorial Front^32,33^. Although the oceanic waters around the archipelago are considered an upwelling system in the equatorial zone^34^, this area is vulnerable to oceanographic disturbances like El Niño events that cause negative fluctuations in marine productivity with resulting demographic impacts on Galapagos otariids^10,22^.

The GSL and GFS are non-migratory species that maintain small rookeries throughout the year, contrary to other pinnipeds in cold zones which aggregate on land mainly during short, highly synchronized breeding seasons^35,36^. It has been shown that in small otariids populations (< 1,300 individuals), all age/sex categories can be identified and counted on land^10^. Since a proportion of the population will be out at sea during the census, estimates of the total population size usually require a correction factor with the count data^8,37^. Given the relevance to conservation efforts, the Galapagos National Park Directorate (GNPD) launched a management plan for the conservation of the GSL in 2012, including a standardized method of counting populations (direct counts in the rookeries during the annual census in the entire archipelago) to determine their population size and to propose appropriate management strategies for this species^29^. All management measures and counting methods proposed for GSL also apply to the GFS populations.

There is a need to generate substantial information to establish the population size and status of the Galapagos otariids in the face of the high environmental variability and growing frequency of ENSO events in the archipelago. Here, we assess the current status of GSL and GFS by analyzing their abundance and population trend based on the counts performed from 2014 to 2018 throughout the archipelago. We estimate the population size of GSL using correction factors applied to the annual censuses^8,10^. We also analyzed the relationships between pup production of both species and oceanographic variables and discuss the possible causes of the variability in these populations.

## Methods

### Study area and data collection

The Galapagos Archipelago is located at 960 km from mainland Ecuador in the Tropical Eastern Pacific. Waters associated with this archipelago make up the Galapagos Marine Reserve (GMR), an area of about 138,000 km^2^ that has protected endemic and native marine species for over 20 years^38^. The region’s oceanographic setting is largely responsible for sporadic colonization of the islands, leading to the evolution and presence of the diverse species such as the Galapagos pinnipeds^31^. The Equatorial Undercurrent and the South Equatorial Current carry high-nutrient waters to the GMR and affect the marine ecosystem dynamics^32,33^. This results in different regions within the archipelago with differentiated marine productivity, sea temperature patterns and biodiversity levels^39,40^. The presence and intensity of these currents in the archipelago determine two seasons throughout the year: a warm season (January to May), and a cold season (June to December), that influence the sea surface temperature (SST), ranging up to eight °C between seasons^41^.

This research was performed as part of the pinnipeds population monitoring program conducted by the GNPD and the Universidad San Francisco de Quito (USFQ). Most of the data were derived from the annual research cruises conducted by the Galapagos Science Center (GSC) in collaboration with the GNPD to assess the impact of climate change on the emblematic fauna of the Galapagos Archipelago. From 2014 to 2018, all breeding rookeries of GSL and GFS were monitored during October, corresponding to the peak in pup births of both species. A total of 43 censuses per year (32 rookeries of GSL and 11 of GFS) were carried out on 13 islands of the archipelago (Fig. 1). Censuses were performed on land using a direct count method commonly employed in otariid surveys^10,42^. Each census started at 6 am and required approximately two to three hours to complete, depending on the size of the rookery. Two trained observers, situated in opposite boundaries of the rookery, walked along the coastline, simultaneously counting animals and identifying them by age/sex categories. The census was completed when the observers encountered each other in the middle of the rookery^10^.

### Data analysis

In this study, the archipelago was divided into four regions (West, North, Central and Southeastern) (Fig. 1), based on the regional biogeography of the archipelago proposed by^39,40^ to identify potential influence of the environmental variability in the Galapagos pinnipeds populations. Six age/sex categories were distinguished in each census: adult males, subadult males, adult females, juveniles, pups and indeterminate (unidentified animals)^42^. Categorized data were organized in a matrix of total counted animals per rookery, bioregion and year (Supplementary Tables S1 and S2).

The GSL abundance in each rookery, island and region were estimated by correcting the number of counted animals using correction factors derived from the probability of observing individuals of different age categories ashore during the counts^8^. These correction factors were obtained by using the Lincoln-Petersen method, based on resights of marked animals, to estimate the population size of GSL in the Caamaño rookery in the central bioregion over 13 years of counts^8^. According to these authors, the probability of observing a given adult in the rookery (*P*_*ob-r*_) during the cold/reproductive season was 16% (95% confidence interval (CI) = 19%–12%), while for juveniles, the probability was 35% (95% CI = 37%–34%). Based on these values, we assumed that the proportion of animals at sea (*p*_*s*_) during the counts was 1/(*P*_*ob-r*_/100). Thus, the numbers of adults and juveniles counted ashore (Ca) were corrected by multiplying Ca * *p*_*s*_. For adults, the *p*_*s*_ values used were 6.25 (95% CI = 5.26–8.33), and for juveniles, the *p*_*s*_ values were 2.86 (95% CI = 2.78–2.94). Individuals categorized as indeterminate in the censuses were considered juveniles for the analyses, as categorization uncertainties mostly concerned the immature category^36^. The sum of the corrected counts of adults and juveniles and the raw counts of pups corresponded to the population estimates in each rookery in a given year.

A regression between years and total individuals counted between 2014 and 2018 was used to estimate the population trends of GSL and GFS in the archipelago. The regression between years and total pups counted was used to estimate the pup abundance trends for the same sites. Census data used in the regressions were natural-logarithm transformed. The value of the slope (r) in the regression analysis was converted to the finite rate of increase (ʎ) as exp (r). The average annual growth rates of the population and of pup abundance, expressed in percentages, were calculated as 100*(ʎ-1). The variability in pup abundance in each rookery throughout the years was determined using the coefficient of variation (CV). Differences in pup abundance per rookery in each region were tested using a Kruskal Wallis test, along with a multiple comparison of the mean ranks for all groups.

### Abundance and environmental variables

The effects on the GSL and GFS pups abundance of oceanographic variables, such as the anomalies in SST (°C) linked to El Niño and La Niña events (measured from the El Niño 1 + 2 index), the subthermocline temperature (ST, °C; between approximately 60 m and 100 m of depth) and the depth-integrated chlorophyll-a concentration (mg m^−2^), a proxy of phytoplankton biomass, were examined per region in the archipelago. For this, census data were natural-logarithm transformed. The values of the El Niño 1 + 2 index were taken from the NOAA website (http://www.cpc.ncep.noaa.gov/data/indices/sstoi.indices). This index is the three months running mean SST anomalies in the region and is commonly used to indicate the status of the equatorial Pacific coasts, including the Galapagos Archipelago. The criterion often used to classify El Niño or La Niña events are five consecutive 3-month running mean SST anomalies exceeding the threshold of ± 0.5 °C.

Temperature and salinity profiles to ~ 100 m depth were obtained using a Seabird SBE 19plus V2 SeaCAT Profiler, collected within two kms offshore of the rookeries. All CTD casts were corrected using SeaBird’s SeaSoft software. Profiles typically exhibited a surface mixed layer and a deep layer of nearly constant properties, separated by a thermocline of varying thickness. The subthermocline temperature (ST) was determined as the average temperature from the bottom of the cast (approximately 100 m) to the depth where the potential density was 0.2 kg/m^3^ less than the density at the bottom of the profile^43^. The upper depth bound averaged from 45–65 m across the years surveyed.

Chlorophyll-a samples (chl *a*) were collected in triplicate by gravity filtering 400 ml of seawater through Isopore 5 µm polycarbonate filters (47 mm) to obtain the large cell size fraction (> 5 µm). The filtrate was then filtered onto a Whatman GF/F filter (25 mm) using an in-line vacuum (≤ 100 mmHg) to obtain the small cell size fraction (≤ 5 µm). The filters were extracted in 6 ml of 90% acetone and incubated in the dark at − 20 ºC for 24 h. Raw fluorescence values of the chl *a* extracts were measured on a Turner Designs 10-AU fluorometer according to the methods of^44^. Depth-integrated total chl *a* was determined through trapezoidal integration of the combined size-fractions at four depth measurements made throughout the euphotic zone corresponding to sampling depths of 50%, 30%, 10% and 1% incident irradiance.

Spearman’s rank correlation coefficient (r_s_) was used to analyze the relationship between the natural logarithm of the total number of pups and the average value (from August to October) of oceanographic variables per year. All statistical analyses were conducted using Statistica version 8.0 (StatSoft. Inc., Tulsa, OK, USA). Statistical significance was defined as p < 0.05.

## Results

### Galapagos sea lions’ abundance and population trend

**Figure 2:**
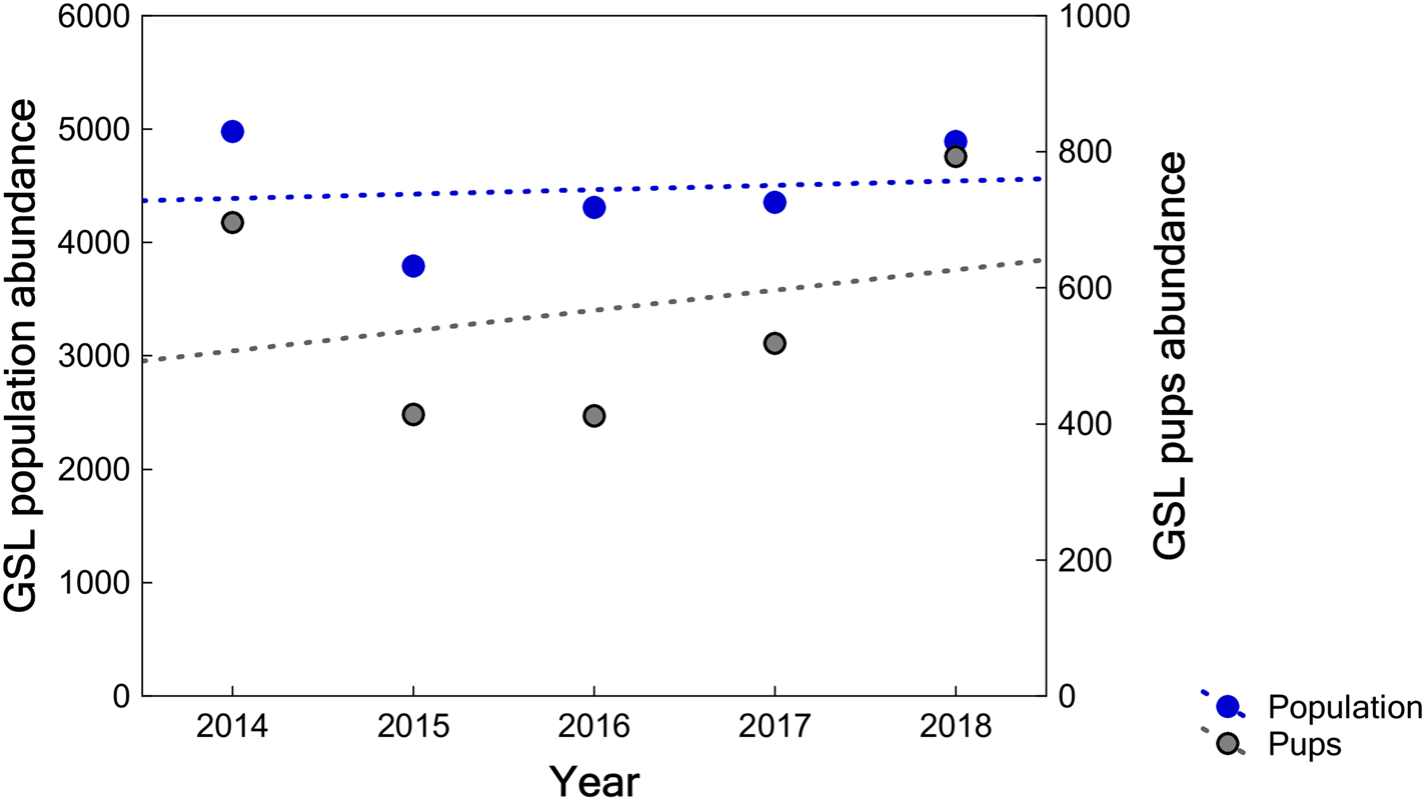
Galapagos sea lions’ population and pup abundance trend in the Galapagos Archipelago from 2014 to 2018. It includes the annual counts at 32 breeding rookeries on 13 islands distributed throughout the archipelago. Blue and gray circles show the total population and pup population, respectively. Blue and gray dashed lines show the population trend and the pup trend, respectively.

From 2014 to 2018, the GSL was recorded at 32 breeding rookeries on 13 islands (Table S1). The estimated population sizes on all islands are shown in Table 1. The 2018 global census resulted in 4,891 individuals counted, representing an estimated 19,929 (95% confidence interval (CI) = 17,617–24,694) animals in total. The greater abundance was estimated in 2014 (21,493; 95% CI = 18,864–26,895 individuals), based on a count of 4,980 animals. Overall, there was a decrease of 23.9% in the number of animals counted from 2014 to 2015 across the archipelago (Fig. 2); whereas from 2015 to 2018 the total of animals counted increased 29%. The average annual growth rate in the archipelago was 1% (slope = 0.01, standard error (SE) = 0.04, p = 0.818, R^2^ = 0.02) between 2014 and 2018 (Fig. 2).

**Table 1:** Galapagos sea lion population sizes in breeding rookeries throughout the archipelago. Estimated values are based on corrected census data using correction factors for different age categories. The 95% confidence interval of each estimate is shown in parentheses.

Region/Island	2014	2015	2016	2017	2018	% Island on Archipelago
**Western** **9.1%**						
Fernandina	1143	1008	1049	886	949	5.3
	(1027–1376)	(882–1267)	(901–1358)	(782–1097)	(837–1181)	
Isabela	1001	749	680	545	777	3.8
	(886–1235)	(665–921)	(607–869)	(489–658)	(691–951)	
Sum Western Region	2144	1757	1729	1431	1726	
	(1914–2611)	(1547–2187)	(1508–2184)	(1271–1755)	(1528–2132)	
**Northern 5.7%**						
Pinta	483	276	428	289	1398	3.1
	(419–615)	(240–350)	(373–541)	(259–351)	(1214–1777)	
Marchena	104	120	30	54	264	0.5
	(91–129)	(108–142)	(27–37)	(49–63)	(232–331)	
Genovesa	544	333	306	447	297	2.1
	(471–697)	(289–427)	(264–393)	(391–562)	(264–365)	
Sum Northern Region	1131	729	764	790	1959	
	(981–1441)	(637–919)	(664–970)	(698–976)	(1710–2472)	
**Central 23.1%**						
Santiago	919	434	623	861	545	3.5
	(812–1136)	(383–537)	(551–770)	(763–1060)	(484–670)	
Rábida	324	169	376	167	636	1.6
	(285–406)	(149–210)	(331–469)	(146–210)	(562–787)	
Seymur	988	650	853	1027	883	4.6
	(880–1206)	(567–820)	(750–1063)	(907–1271)	(782–1090)	
Santa Cruz	2825	1908	2788	2665	2561	13.4
	(2459–3583)	(1688–2358)	(2434–3518)	(2354–3301)	(2273–3148)	
Sum Central Region	5056	3161	4640	4720	4626	
	(4435–6331)	(2788–3925)	(4067–5820)	(4171–5842)	(4101–5696)	
**Southeastern 62.1%**						
Santa Fe	1289	1017	1943	1432	1395	7.4
	(1130–1617)	(903–1249)	(1705–2433)	(1282–1732)	(1255–1675)	
Floreana	3682	2487	2785	3182	3070	15.9
	(3213–4651)	(2176–3126)	(2433–3510)	(2786–3997)	(2708–3811)	
Española	1981	1081	1357	1293	1533	7.6
	(1752–2450)	(973–1297)	(1194–1691)	(1136–1615)	(1374–1855)	
San Cristóbal	6210	6362	5935	5647	5619	31.2
	(5441–7794)	(5555–8022)	(5239–7354)	(4969–7035)	(4942–7009)	
Sum Southeastern Region	13,162	10,947	12,021	11,554	11,617	
	(11,534–16,512)	(9608–13,695)	(10,571–14,988)	(10,173–14,379)	(10,278–14,350)

During the study period, larger rookeries were located in the southeastern region (62.0 ± 2.4% of the entire population), following by the central region (23.5 ± 2.3%), the western region (9.6 ± 1.4%), and the northern region (5.0 ± 2.0%). The populations in the central and northern rookeries increased at an average annual growth rate of 4.5% (slope = 0.04, SE = 0.11, p = 0.531, R^2^ = 0.14) and 15.3% (slope = 0.14, SE = 0.13, p = 0.341, R^2^ = 0.30) respectively (Fig. S1). The populations in the western and southeastern regions decreased at an average rate of -6.6% (slope = -− 0.07, SE = 0.05, p = 0.291, R^2^ = 0.35) and − 0.3% (slope = -0.01, SE = 0.03, p = 0.919, R^2^ = 0.01) respectively, between 2014 and 2018 (Fig. S1).

During the five years of sampling, the annual average (± standard deviation, SD) number of pups was 567 ± 153 individuals. However, pup abundance varied across years in the archipelago, where the highest and the lowest interannual variability were observed in the northern (average coefficient of variation (CV) = 82.5%) and central (CV = 33.4%) rookeries, respectively (Table 2). The pup abundance in the archipelago decreased 40.5% between 2014 and 2016, whereas from 2015 to 2018 it increased 91.5%. The average annual growth rate of pup abundance was 5% between 2014 and 2018 (slope = 0.05, SE = 0.11, p = 0.677, R^2^ = 0.07) (Fig. 2). This increasing pup trend between 2014 and 2018 was observed in northern and central rookeries at an average annual rate of 100.3% (slope = 0.69, SE = 0.22, p = 0.050, R^2^ = 0.77) and 12.9% (slope = 0.12, SE = 0.07, p = 0.183, R^2^ = 0.50), respectively. In the west, pups decreased at an average rate of − 6.7% (slope  =  − 0.07, SE = 0.15, p = 0.677, R^2^ = 0.07) between 2014 and 2018; whereas in the southeastern, the average annual growth rate is 3.1% (slope = 0.03, SE = 0.12, p = 0.898, R^2^ = 0.02).

**Table 2:** Galapagos sea lion pups counted in breeding rookeries throughout the archipelago. The coefficient of variation (CV) shows the variability in the number of pups counted per island throughout the years.

Region/Island	2014	2015	2016	2017	2018	CV
**Western**						
Fernandina	75	23	27	19	63	0.62
Isabela	29	21	24	18	17	0.22
**Northern**						
Pinta	1	0	2	3	5	0.87
Marchena	0	0	0	0	2	2.24
Genovesa	0	7	1	12	15	0.94
**Central**						
Santiago	33	25	24	32	40	0.21
Rábida	4	5	2	2	10	0.71
Seymur	43	27	23	26	46	0.32
Santa Cruz	60	57	62	107	116	0.36
**Southeastern**						
Santa Fe	30	34	27	67	78	0.50
Floreana	62	58	38	72	97	0.33
Española	107	66	62	47	86	0.32
San Cristóbal	252	91	120	113	218	0.45

The number of pups in 2018 was 793 individuals, which was the highest value during the five years. Fifty percent of births occurred in the southeastern rookeries, 37% in the central, 10% in western, and 8% in northern rookeries. There were significant differences between years (Kruskal–Wallis test: H_(4)_ = 13.66, p = 0.008) and regions (Kruskal–Wallis test: H_(3)_ = 16.55, p = 0.001) in the numbers of pups counted from 2014 to 2018. A multiple comparisons test revealed that the numbers of pups counted in 2015 differed significantly from 2017 and 2018 (p < 0.05), and the numbers of pups at northern rookeries differed significantly from the central and southeastern rookeries in all years (p < 0.05).

### Galapagos fur seals’ abundance and population trend

**Figure 3:**
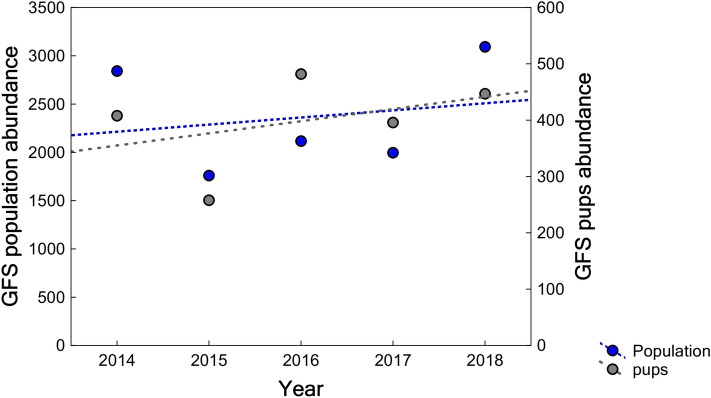
Galapagos fur seals’ population and pup abundance trend in the Galapagos Archipelago from 2014 to 2018. It includes the annual counts at 11 breeding rookeries on seven islands distributed in the western, northern and central regions of the archipelago which this species inhabits. Blue and gray circles show the total population and pup population, respectively. The blue dashed line shows the population trend.

From 2014 to 2018, the GFS was recorded at 11 breeding rookeries on seven islands (Table S2). The 2018 global census was the largest, resulting in 3,093 individuals counted. Overall, there was a 38.1% reduction between 2014 and 2015 in the number of animals counted, followed by a 78.7% increase between 2015 and 2018 (Fig. 3). The average annual growth rate in the archipelago was 3% (slope = 0.03, SE = 0.09, p = 0.755, R^2^ = 0.04) between 2014 to 2018 (Fig. 3).

During the study period, larger rookeries were located in the western region (77.9 ± 4.8% of the entire population), following by the northern region (18.0 ± 4.7%), and the central region (4.1 ± 0.4%). The average annual growth rate in the western rookeries was 3.3% (slope = 0.03, SE = 0.10, p = 0.772, R^2^ = 0.03) between 2014 to 2018, while for the northern and central rookeries it was 3.9% (slope = 0.04, SE = 0.07, p = 0.637, R^2^ = 0.08) and −0.2% (slope = −0.01, SE = 0.07, p = 0.975, R^2^ = 0.01) respectively (Fig. S2).

During the five years of sampling, the annual average (± SD) number of pups was 398 ± 76 individuals, and the highest and the lowest interannual variability were observed in the central (CV = 50.3%) and western (CV = 18.3%) rookeries, respectively (Table 3). The pup abundance throughout the archipelago decreased by 36.8% between 2014 and 2015, whereas from 2015 to 2016, it increased by 86.8% due to a tripling in total pups in the northern region (Fig. 3). The number of pups increased between 2014 and 2018 in the archipelago at an average annual rate of 6.3% (slope = 0.06, SE = 0.08, p = 0.508, R^2^ = 0.16) (Fig. 3). This trend was observed in western, northern and central rookeries at an average annual rate of 5.7% (slope = 0.06, SE = 0.08, p = 0.524, R^2^ = 0.15), 7.6% (slope = 0.07, SE = 0.11, p = 0.641, R^2^ = 0.08) and 20.9% (slope = 0.19, SE = 0.11, p = 0.174, R^2^ = 0.51), respectively.

**Table 3:** Galapagos fur seal pups counted in breeding rookeries of the archipelago. The coefficient of variation (CV) shows the variability in the number of pups counted per island throughout the years.

Region/Island	2014	2015	2016	2017	2018	CV
**Western**						
Fernandina	303	178	334	310	264	0.22
Isabela	66	54	76	48	128	0.43
**Northern**						
Pinta	26	19	57	27	29	0.46
Marchena	4	0	4	2	7	0.77
Genovesa	0	1	0	0	0	2.24
**Central**						
Santiago	7	6	10	9	10	0.22
Rábida	2	0	1	0	9	1.58

The number of pups counted in 2018 was 447 individuals, which was the second highest value during the five years. Eighty-eight percent of pup’s production occurred in the western rookeries, 8% in northern and 4% in central rookeries. There were no significant differences between years in the numbers of pups counted from 2014 to 2018 (Kruskal–Wallis test: H_(4)_ = 1.39, p = 0.845), but differences were observed between regions (Kruskal–Wallis test: H_(2)_ = 12.52, p = 0.001). A multiple comparisons test revealed that the number of pups at western rookeries differed significantly from the number in the central rookeries (p < 0.05).

### Environmental variables and regional pup abundance

The average (± standard deviation) values of ST and chl *a* (from August to October) recorded from 2014 to 2018 per island in each region are shown in Table 4. There were no significant relationships between the oceanographic variables and the GSL and GFS pup abundances in any region of the archipelago (Tables 5, 6). Pup abundance of GSL showed a slight negative trend with the El Niño 1 + 2 index and ST values in most regions, and a positive trend with the depth-integrated chl *a* data in the western and southeastern regions (Table 5). While the pup abundance of GFS showed a negative trend with the El Niño 1 + 2 index and ST values, and a positive trend with the depth-integrated chl *a* data in all regions (Table 6).

**Table 4:** Subthermocline temperature (ST, °C) and depth-integrated chlorophyll-a concentration (Chl *a*, mg*m^−2^) values (means ± SD), recorded in areas near the main rookeries of Galapagos sea lions and Galapagos fur seals between 2014–2018.

Region/Island	2014		2015		2016		2018	
	ST (°C)	Chl *a* (mg*m^−3^)	ST (°C)	Chl *a* (mg*m^−3^)	ST (°C)	Chl *a* (mg*m^−3^)	ST (°C)	Chl *a* (mg*m^−3^)
**Western**								
Fernandina	15.88 ± 0.23	48.27 ± 16.79	18.38 ± 0.16	27.60 ± 1.26	14.87 ± 0.29	39.92 ± 15.51	17.01 ± 0.79	22.22 ± 9.26
Isabela	15.40 ± 0.28	31.41 ± 20.53	18.14 ± 0.45	14.18 ± 0.85	15.46 ± 0.29	34.17 ± 7.57	15.88 ± 0.05	25.45 ± 1.46
**Northern**								
Pinta	15.73 ± 0.13	19.38 ± 2.45	19.19 ± 0.38	15.28 ± 0.67	20.42 ± 1.34	33.82 ± 3.17	15.71 ± 0.29	14.07 ± 0.45
Marchena	15.92 ± 0.45	–	20.91 ± 0.67	14.44 ± 0.96	14.81 ± 0.23	26.49 ± 2.87	15.61 ± 0.46	14.74 ± 0.22
Genovesa	20.23 ± 0.54	60.68 ± 18.25	20.42 ± 0.12	33.82 ± 9.52	14.53 ± 0.45	18.38 ± 5.25	17.26 ± 0.17	36.83 ± 0.38
**Central**								
Santiago	15.34 ± 0.23	41.01 ± 3.67	17.37 ± 0.23	35.60 ± 8.24	14.64 ± 0.18	34.80 ± 11.18	15.38 ± 0.52	15.14 ± 0.18
Rábida	15.75 ± 0.54	–	19.46 ± 0.44	–	14.58 ± 0.29	–	16.01 ± 0.84	–
Seymur	15.72 ± 0.12	–	18.26 ± 0.25	–	14.87 ± 0.45	–	15.36 ± 0.45	–
Santa Cruz	15.66 ± 0.25	50.55 ± 9.33	18.42 ± 0.18	16.18 ± 0.36	14.88 ± 0.32	19.55 ± 3.51	15.31 ± 0.28	21.11 ± 0.32
**Southeastern**								
Santa Fe	17.37 ± 0.32	–	17.37 ± 0.34	–	14.79 ± 0.56	–	15.67 ± 0.67	–
Floreana	16.03 ± 0.57	41.01 ± 1.83	18.04 ± 0.42	15.52 ± 0.21	15.19 ± 0.21	23.89 ± 1.98	16.53 ± 0.21	12.07 ± 0.13
Española	17.22 ± 0.43	17.07 ± 1.49	17.56 ± 0.23	17.92 ± 0.95	15.00 ± 0.18	25.53 ± 1.35	15.79 ± 0.34	27.67 ± 0.34
San Cristóbal	15.95 ± 0.27	19.92 ± 2.05	17.98 ± 0.13	17.64 ± 0.76	14.88 ± 0.07	18.88 ± 2.45	15.44 ± 0.05	23.81 ± 0.26

*There are no available data for 2017.

**Table 5:** Spearman’s rank correlation coefficient results. Relationships between Galapagos sea lion pup abundance per region of the archipelago and environmental variables from 2014 to 2018 for Niño 1 + 2 index and without 2017 data for subthermocline temperature (ST) and Chlorophyll-a (Chl *a*)

Region	Variables	Spearman rank correlation coefficient result
**Western**		
Pup abundance vs	Niño 1 + 2 index	r_s_ = 0.30
	ST	r_s_ = − 0.40
	Chl *a*	r_s_ = 0.40
**Northern**		
Pup abundance vs	Niño 1 + 2 index	r_s_ = − 0.60
	ST	r_s_ = 0.00
	Chl *a*	r_s_ = − 1.00
**Central**		
Pup abundance vs	Niño 1 + 2 index	r_s_ = − 0.60
	ST	r_s_ = 0.20
	Chl *a*	r_s_ = − 0.20
**Southeastern**		
Pup abundance vs	Niño 1 + 2 index	r_s_ = − 0.30
	ST	r_s_ = − 0.20
	Chl *a*	r_s_ = 0.20

No relationship was significant at p = 0.05.

**Table 6:** Spearman’s rank correlation coefficient results. Relationships between Galapagos fur seal pup abundance per region of the archipelago and environmental variables from 2014 to 2018 for Niño 1 + 2 index and without 2017 data for subthermocline temperature (ST) and Chlorophyll-a (Chl *a*).

Region		Variables	Spearman rank correlation coefficient result
**Western**			
Pup abundance vs		Niño 1 + 2 index	r_s_ = − 0.40
		ST	r_s_ = − 0.80
		Chl *a*	r_s_ = 0.40
**Northern**			
Pup abundance vs		Niño 1 + 2 index	r_s_ = − 0.40
		ST	r_s_ = − 1.00
		Chl *a*	r_s_ = 1.00
**Central**			
Pup abundance vs		Niño 1 + 2 index	r_s_ = − 0.46
		ST	r_s_ = − 0.40
		Chl *a*	r_s_ = − 1.00

No relationship was significant at p = 0.05.

**Figure 4:**
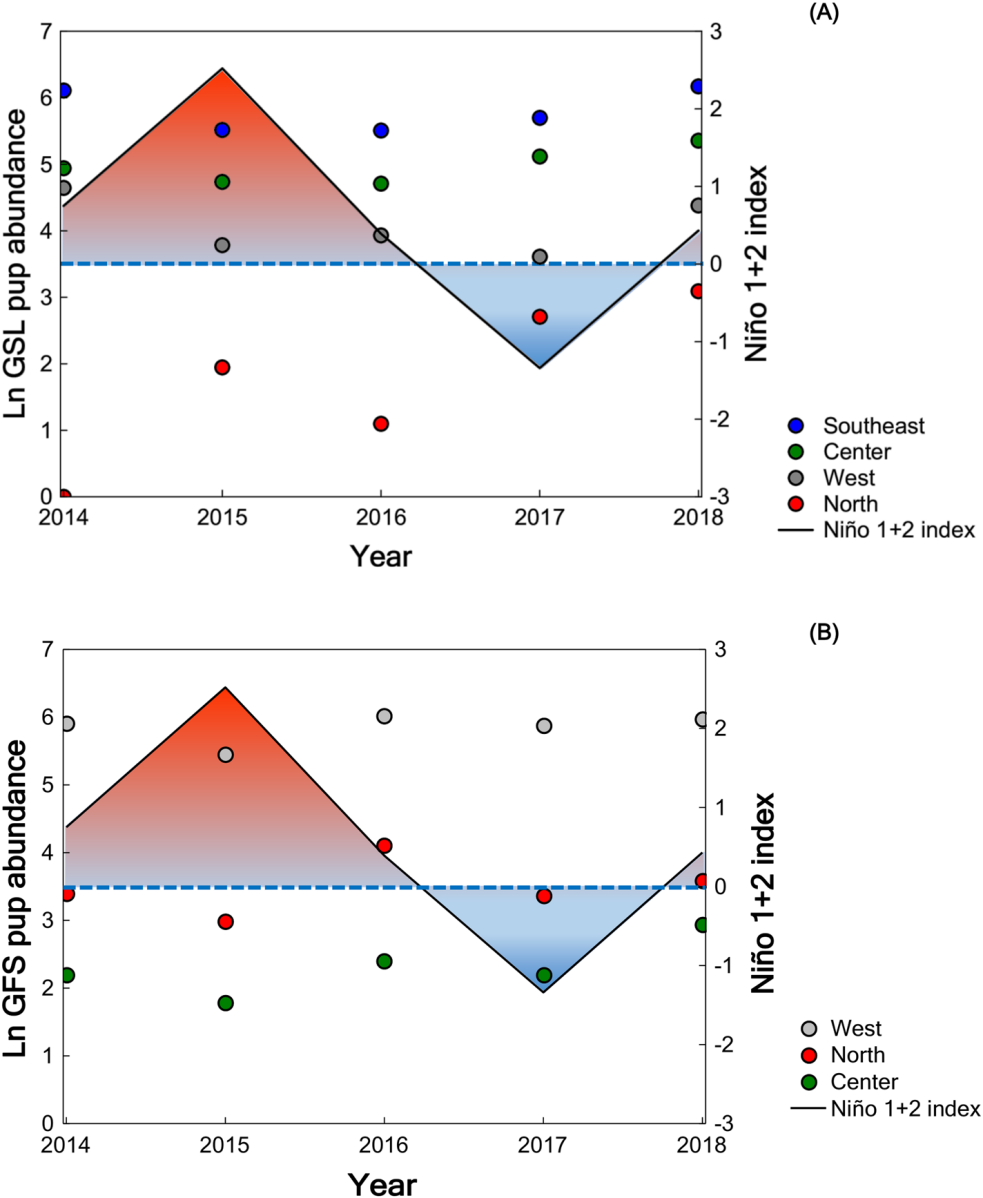
Annual pup abundance per region of the Galapagos Archipelago concerning the El Niño 1 + 2 index. (**A**) Galapagos sea lion. (**B**) Galapagos fur seal. The El Niño 1 + 2 index is the three-month running average of the sea surface temperature (SST) anomalies in the El Niño 1 + 2 region (0–10°S, 90–80°W). The values of the El Niño 1 + 2 index corresponding to October (during the peak in pup births of both species) were plotted for each year and taken from the NOAA website. The Niño El 1 + 2 index indicates the intensity of El Niño (positive values) and La Niña (negative values) events, which can be considered weak (SST anomaly of 0.5 to 0.9), moderate (1.0 to 1.4) or, strong (> 1.5).

In the GSL, the tendency with the El Niño 1 + 2 index is stronger in the north and central regions, less evident in the southeastern region and was not observed in the west (Fig. 4A). The relationships between GSL pup abundance and ST and chl *a* were as expected (negative with ST and positive with chl *a*) in the west and southeastern regions, but contrary in the north and central regions. In the GFS, the negative relationships between pup abundance and the El Niño 1 + 2 index (Fig. 4B) and ST values were evident in the three regions which this species inhabits. However, the positive relationship between GFS pup abundance and chl *a* was evident in the north and west but not in the central region.

## Discussion

Determining the abundance and population trends of Galapagos pinnipeds is a complex task that depends on the assumptions of techniques through which the census is performed. However, in these polygynous pinnipeds, breeding seasons occur annually within a limited, predictable timeframe^35^. Therefore, pup production is believed to be the best indicator of rookery status and from which the population trends can be estimated^6,11^. We report estimates of GSL and GFS population abundances during their reproductive peaks, derived from a direct count method commonly employed in Galapagos otariids^8,10^, that provides estimated values with narrow confidence intervals.

### Galapagos sea lion’s population trend

During the last decades, the Galapagos pinnipeds have experienced significant population declines^10,26^. Therefore, both species are protected by Ecuadorian laws, and its natural environment is managed under conservation plans^29^. The current size of the GSL population was estimated at ~ 20,000 individuals and increased at an average annual rate of 1% between 2014 and 2018. For the population estimate, we use correction factors calculated from GSL observations in a central rookery. However, it is important to consider that there may be differences in the phenology of births between regions that might affect the proportion of animals on land during the counts^8^. The population peaked in 2014, followed by a population decline of 23.8% in 2015, which was associated with the strong El Niño event of that year. Since the abundance and distribution of pinnipeds are influenced by their ability to feed while at sea^45,46^, oceanographic factors seem to be the main causes of population fluctuations.

Recent population estimates differ from the first census in 1978, probably reflecting differences in the methods used. In 1978, a direct and at-distance count of 8,000 animals suggested an estimated population of 40,000 individuals^30^, while in 2001, the direct and at-distance count was 4,937 sea lions, resulting in an estimated 16,000 individuals^26^. As the rationales behind the correction factors were not provided, these estimates may not be compared. Since 2012, counts have followed a standardized method based on the management plan for Galapagos sea lion’s conservation^29^. As animal counts for 2018 remain close to that recorded in 2001 and that the average age of reproducing individuals is ~ 10 years with a typical lifespan ≥ of 18 years^47^, the population may have been relatively stable over the past 20 years. If so, this suggests that a population reduction of 50% is maintained in the last four generations (1978–2018), which would ratify its classification as an endangered species^27^. Thus, the GSL maintains residual effects of the strong 1982/83 and 1997/98 El Niño events^22,26,48^ from which they have not been able to recover due to the recurrence of El Niño events every 4–5 years^49,50^.

The GSL breeds on almost all the islands of the archipelago and approximately 62% of the population inhabit the southeastern region. The El Malecón rookery, on San Cristobal Island, is the largest in the archipelago (Table S1) and maintains an annual increase close to 2% reported by^10^, during favorable oceanographic conditions. After an El Niño event, the GSL populations increase mainly in the central and southeastern rookeries, while northern and western rookeries maintain a reduced population for a longer period. This is explained by the marine habitat characteristics and the population dynamics, which differ in each region of the Galapagos Archipelago^51,52^, influencing the abundance of the GSL and determining the population trends of its rookeries. The characteristics of shelf habitat could lead to a restriction of food resources^25^. The northern region has few areas where benthic habitat is attainable to GSL, which causes them to increase their feeding effort^53^, making this region a more demanding environment for GSL, reflected in its population size. Oceanographic conditions show upwellings in the western region^32^. These high levels of marine productivity could favor the trophic requirements of the GSL and, consequently, their population growth. However, in this region, the GSL limits its foraging effort and probably its population size to reduce competition with GFS^24,25^, which has significantly larger populations. Therefore, the slow population recovery in the northern and western regions could be associated with ecological conditions, their rookeries’ size and with the low pup production that is maintained. These conditions confirm that the population trend of a single region or island should not be considered representative of the entire archipelago^8,10^.

Pup abundance varied across years throughout the archipelago, decreasing about 40% during the El Niño event, and increased at an average annual rate of 5% between 2014 and 2018. These trends were maintained in the southeastern rookeries, while in other regions, the recovery rate was lower. This suggests that northern and western regions are less important as a breeding rookery, and rather these sites could be used as haul-out areas for GSL. The breeding success and growth of rookeries around the archipelago are linked to feeding resources available for mothers^54–56^. Therefore, a low trophic efficiency related to a different diet in females from the northern and western regions could be reflected in the annual pup abundance. There are spatial differences in the GSL diet that suggests the presence of specific foraging areas with distinct prey components for each region^53^. The western region has the greatest diversity of prey species, however, low-calorie fish as myctophids are their main prey. While the main prey in the central and southeastern regions are larger fish of better caloric quality, such as sand bass and mottled scorpionfish^53,55^.

### Galapagos fur seal’s population trend

GFS populations have undergone a drastic decline related to inter-annual warming events and anthropogenic stressors, such as the 19th-century commercial sealing operations in the Galapagos Archipelago^28,57^. Like GSL, its population peaked in 2014 with a 38% decline in 2015 during the strong El Niño event conditions. The total population in the archipelago has always been low in comparison to GSL, however, these conditions are not maintained in sympatric rookeries as Cabo Hammond and Cabo Douglas, where the number of GFS is approximately five times that of GSL. The potential trophic overlap between GFS and GSL seems to be linked to environmental variability since these otariids tend to overlap in their foraging zones during inter-annual warm events such as El Niño^24^. These conditions could favor the GFS populations, since being the most abundant in the western region, they would easily displace the GSL populations. This behavior has already been observed on the coasts of Uruguay, where the South American fur seal (*Arctocephalus australis*) is the most abundant species, a product of a gradual displacement of the South American sea lion (*Otaria flavences*), that has significantly decreased its population^58^.

Most individuals are concentrated on a few rookeries in the western and northern islands of the archipelago, with 76.7% of the population found in Fernandina and Isabela islands. Western islands are situated in a region of strong upwelling and high productivity^33,34^, suggesting a geostrophic association to this hotspot within a tropical environment in the equatorial Pacific Ocean^24,25^. The GFS is the smallest of all otariids and exhibits an unusually restricted geographic range for a pinniped^23,59^, accompanied by high fidelity to its breeding rookeries that might even have reduced their genetic variability^60^. The link between high levels of philopatry and resource availability in otariids could exert strong geographical effects on its population dynamics^45,46^. It is then possible that the regional population trends of GFS are related to high-quality habitat and greater availability of resources^24,61^.

Pup abundances varied across years, decreasing about 36.7% during the El Niño event. The average annual growth rate was calculated in 6.3% between 2014 and 2018. This trend was maintained in western rookeries, while in other regions the recovery rates were lower, suggesting that the central and northern regions are less important as breeding rookeries and that these sites could be recolonization areas. There are disproportionately large populations in the western region concerning central and northern regions. The Santiago Island population in the central region is the smallest rookery, and apparently, it is a rookery which has been stable for the past two decades. Likewise, the Pinta Island population in the north region constituted 14% of the entire Galapagos population and showed an important population increase between 2001 and 2018. These results indicate the most significant increase was from 2001 (40 individuals counted^26^) to 2014–18 (an average of 313 individuals counted), making it the third-largest rookery to date. A dispersal of individuals from the western region to Pinta Island to escape crowding and local competition could explain this population increase. Population dispersions motivated by density-dependent elements have been reported in other pinnipeds as a strategy to facilitate their population growth^62,63^.

The last population monitoring carried out in 2002 indicated a reduction of more than 60% in a period of 24 years relative to counts from 1978^26,30^. However, these results are not the best estimates of GFS abundance^28^. Our results demonstrate GFS populations seem to have remained stable over the past few decades. The average count for 2014 to 2018 is close to the count in 2001, suggesting minimal changes in abundance over the last 20 years. Age structure data are not available for GFS. However, based on information from other *Arctocephalus*, the average age of reproduction may be estimated anywhere between 9 and 12 years of age, with maximum longevity of 20 years^64,65^. This shows that the GFS population maintains a 50% reduction during the last four generations (1978–2018), which suggests the conservation status of this species should remain Endangered. This important population decline may also be associated with the impacts of strong El Niño events in 1982/83 and 1997/98, which caused acute shortages of prey and high rates of mortality^48,66^.

From the management perspective, there is a great interest to know the GFS population size. Unfortunately, there is little information on the population dynamics of this species that allows estimating specific correction factors for the counts on land. As an alternative, the GSL correction factors could be applied in the GFS counts taking into account that both pinnipeds display certain similarities in their life histories. For example, both otariids exhibit high fidelity to their breeding rookeries^10,26,30^ and feeding grounds^55,61^, show similarities in the maternal care and reproductive strategies^35,67,68^ and other adaptation strategies to the Galapagos environment^69^. In this way, the current population size of GFS was estimated at ~ 13,000 individuals (Table S3). However, we do not rule out the possibility of over-or underestimates of the GFS population as the correction factors to counts are not species-specific. Thus, further studies are needed to improve our knowledge and the accuracy of these estimates.

### Oceanographic conditions and pup abundance

Galapagos pinnipeds reflect short-term changes in response to inter-annual warm events such as the El Niño event^22,48^, which generate a strong depletion of main prey availability and changes in foraging patterns^24,66^. The oceanographic conditions recorded in the Galapagos Archipelago show that the effects of the El Niño event on the population dynamics of GSL and GFS tend to remain for at least the next two years, and after this time there is a notable recovery. Both species are top predators in the region^24,51^, therefore, long-term warming would produce a progressive habitat change affecting multiple generations^70^. California sea lions (*Zalophus californianus*) and Guadalupe fur seals (*Arctocephalus townsendi*) also showed a decline in the pups’ production during the El Niño event 2014–2015, which, in both otariids, was associated with nutritional stress in females and the potential impact on reproductive success^15^. While northern elephant seals (*Mirounga angustirostris)* populations of Baja California show a decline in their abundance because the animals are not migrating as far south to avoid thermal stress (metabolic rate increases) that is associated with warming sea and its effect on air temperature^71^. Thus, it is essential to establish accurate population trends in pinnipeds during the presence of anomalous oceanographic events to predict potential changes under climate change scenarios.

The interannual variability in GSL pup abundance showed a positive relationship with the chlorophyll-a values and a negative relationship with the subthermocline temperature values in western and southeastern regions. Both environmental variables averaged over the period from August to October in each year. However, unexpectedly the northern and central regions showed an inverse relationship with both variables, which could be due to the oceanographic and demographic characteristics of these regions. The northern region is characterized by having a slight influence of the South Equatorial Current and Panama Bight Cyclonic Gyre that causes a low marine production^32^, while the central region is considered a mixing area^33^. Both conditions limit the resilience of GSL females since after anomalous oceanographic events (such as El Niño) they usually need between one and two years to regain their reproductive synchrony^47,72^. While the upwellings of the western region^33^, and the greater abundance of females in the southeast region^10^ could compensate or facilitate the generation of new cohorts.

The GFS pup abundance showed a positive relationship with the chlorophyll-a values and a negative relationship with the subthermocline temperature values in all regions, except for the central region that showed a negative relationship with the chlorophyll-a values. This discrepancy could be explained by the limited GFS population in the only rookery located in the central region, a condition that would decrease the birth rate of the species. Climate variability strongly influences marine productivity, with repercussions in the trophic food web all the way up to top predators^73,74^. Thus, chlorophyll-a is highly correlated with other variables, such as temperature in the ocean^33,34^, and is a good indicator of the climate variability that affects the reproductive success of Galapagos pinnipeds and the abundance of their prey^10,75^. GSL and GFS exhibited seasonal fluctuations according to the availability of their main prey^66,75^. This flexibility in the trophic behavior has enabled them to adapt to the changing conditions of the Galapagos Archipelago and thereby improve their survival^55,76^.

The Galapagos Archipelago is a region where levels of marine productivity are unpredictable compared to other upwelling systems at higher latitudes^77,78^. This creates an ecological challenge for Galapagos pinnipeds, so their population trend changes could be associated with a diet change^79,80^. In years of unusually good feeding conditions, females apparently regulate feeding efforts by staying ashore longer than by shortening their foraging trips^67,68^. In contrast, during anomalous low-prey conditions (El Niño years), females extend their trips to the sea, abandoning their pups and increasing mortality in this age category^35,48^. The presence of El Niño conditions also impacts California sea lion and South American sea lion (*Otaria flavences*) populations modifying main prey availability, increasing foraging effort, and thus decreasing the number of time females spend on land, which causes a negative effect on pups’ body condition and survival^14,15^. The GSL and GFS are highly philopatric species and restrict their foraging trips to areas located within 70 km from the coast^25,61^. While GSL shows a trophic specialization accompanied by different foraging strategies and different prey^55,81^, during ENSO events, some overlap may occur in GSL foraging zones, accompanied by a change of foraging strategies and main prey choices^75^.

The energetic costs of living in a warm climate with lower resource availability may have selected for a reduction in metabolic rate in other otariids^59^. This highlights the fundamental role of the physical environment in shaping the physiology of these species^56,59^. Reduced prey availability in El Niño years causes females to increase their foraging effort (more time at sea), so the pups receive less energy and their survivability decreases^14,48^. There is a negative relationship between sea temperature levels and the early development of GSL pups, thus, during warm conditions, there are smaller pups with reduced weight^36,82^. Both species are among the otariids with the longest lactation periods (2–3 years^35^). The extension of maternal investment in these otariids is a response mechanism to increase the chances of pup survival when populations are exposed to food stress due to drastic changes in oceanographic conditions^35,83^.

## Conclusions and recommendations

The monitoring of Galapagos pinnipeds between 2014 and 2018, incorporating annual population-wide surveys, has recorded short-term changes in response to inter-annual warming events such as the El Niño event. It is understood that there are multiple reasons for monitoring these endangered species. This work highlights key parameters that need to be measured to understand population trends, while also providing up-to-date information of their abundance to assist with the prioritizing of rookeries. Our results show that population information of both species may have localized relevance. Therefore, adequate protection probably requires that each rookery be managed according to its specific conditions. For example, rookeries identified as important breeding sites should receive additional protection because they may become source populations for other rookeries. The Galapagos pinniped monitoring program must continue providing data to help local resource managers make effective decisions. Spatial abundance data and knowledge of population trends allow establishing effective protected areas and facilitate wildlife management. Complementary research programs investigating the feeding patterns, health status, and pup development need to be coordinated, along with demographic information to understand the population dynamics of Galapagos pinnipeds. Finally, we recommend that the potential funds designed for monitoring be maintained over time and that the global census be continued in the long-term to accurately predict the GSL and GFS population trends in the archipelago.

## Supplementary Information

Supplementary Information
