# Supplementary material for: Declines and recovery in endangered Galapagos pinnipeds during the El Niño event
Source: Sci Rep. 2021 Apr 22;11:8785. doi: 10.1038/s41598-021-88350-0 (PMC8075323; doi:10.1038/s41598-021-88350-0)

***Supplementary information***

**Declines and recovery in endangered Galapagos pinnipeds during the El Niño event**

*Diego Páez-Rosas^1^, Jorge Torres^2^, Eduardo Espinoza^2^, Adrian Marchetti^3^, Harvey Seim^3^, Marjorie Riofrío-Lazo^1^

*^1^Universidad San Francisco de Quito, Galapagos Science Center. Isla San Cristóbal, Islas Galápagos, Ecuador.*

*^2^Dirección Parque Nacional Galápagos, Departamento de Ecosistemas Marinos. Islas Galápagos, Ecuador.*

*^3^The University of North Carolina at Chapel Hill, Department of Marine Sciences. Chapel Hill, NC, United States.*

***Corresponding Author:**

Dr. Diego Páez-Rosas

Universidad San Francisco de Quito.

Isla San Cristóbal, Galápagos, Ecuador.

Email: [dpaez@usfq.edu.ec](mailto:dpaez@usfq.edu.ec)

**Table S1.** Total counts (2014–2018) of Galapagos sea lion in breeding rookeries throughout the archipelago.

| **Island** | **Rookery** | **2014** | **2015** | **2016** | **2017** | **2018** | **% Rookery on island** | **% Rookery on archipelago** |
| --- | --- | --- | --- | --- | --- | --- | --- | --- |
| ***Western region*** |  |  |  |  |  |  |  |  |
| Fernandina (5.40%) | Cabo Hammond | 16 | 8 | 7 | 5 | 4 | 3.18 | 0.18 |
|  | Punta Mangle | 215 | 150 | 144 | 117 | 168 | 65.49 | 3.55 |
|  | Cabo Douglas | 26 | 23 | 24 | 39 | 43 | 13.25 | 0.69 |
|  | Punta Espinoza | 66 | 43 | 35 | 45 | 30 | 18.08 | 0.98 |
|  | *Sum Fernandina* | *323* | *224* | *210* | *206* | *245* |  |  |
| Isabela (4.16%) | Pta. Vicente Roca | 17 | 26 | 27 | 15 | 49 | 14.70 | 0.60 |
|  | Pta. Albermale | 58 | 29 | 12 | 29 | 15 | 15.06 | 0.64 |
|  | Cabo Marshall | 45 | 20 | 13 | 24 | 5 | 11.34 | 0.48 |
|  | Lobería/Tintoreras | 121 | 107 | 122 | 74 | 119 | 58.90 | 2.44 |
|  | *Sum Isabela* | *241* | *182* | *174* | *142* | *188* |  |  |
| ***Northern region*** |  |  |  |  |  |  |  |  |
| Pinta (2.55%) | Cabo Chalmers | 36 | 28 | 41 | 26 | 104 | 42.05 | 1.03 |
|  | Cabo Ibettson | 60 | 27 | 47 | 44 | 177 | 58.54 | 1.53 |
|  | *Sum Pinta* | *96* | *55* | *88* | *70* | *281* |  |  |
| Marchena (0.58%) | Punta Calle | 22 | 30 | 7 | 14 | 57 | 100 | 0.58 |
| Genovesa (1.83%) | Bahía Darwin | 105 | 69 | 59 | 100 | 77 | 100 | 1.83 |
| ***Central region*** |  |  |  |  |  |  |  |  |
| Santiago (3.78%) | Puerto Egas | 98 | 40 | 82 | 70 | 97 | 39.29 | 1.70 |
|  | Sombrero chino | 125 | 71 | 71 | 143 | 51 | 49.87 | 2.07 |
|  | *Sum Santiago* | *223* | *111* | *153* | *213* | *148* |  |  |
| Rábida (1.64%) | Playa Roja | 71 | 40 | 82 | 36 | 146 | 100 | 1.64 |
| Seymur (4.75%) | Seymur Norte | 158 | 24 | 91 | 102 | 79 | 40.33 | 1.98 |
|  | Mosquera | 97 | 126 | 106 | 140 | 146 | 59.94 | 2.79 |
|  | *Sum Seymur* | *255* | *150* | *197* | *242* | *225* |  |  |
| Santa Cruz (13.33%) | Plazas | 322 | 237 | 361 | 357 | 340 | 54.24 | 7.25 |
|  | Caamaño | 283 | 221 | 249 | 295 | 310 | 45.76 | 6.08 |
|  | *Sum Santa Cruz* | *605* | *458* | *610* | *652* | *650* |  |  |
| ***Southeastern region*** |  |  |  |  |  |  |  |  |
| Santa Fe (7.81%) | Bahía Santa Fe | 289 | 251 | 427 | 382 | 388 | 100 | 7.81 |
| Floreana (15.10%) | Post Office | 215 | 293 | 229 | 229 | 156 | 34.46 | 5.16 |
|  | Champion | 130 | 86 | 201 | 189 | 383 | 28.98 | 4.34 |
|  | Las Cuevas | 383 | 144 | 107 | 195 | 129 | 27.54 | 4.22 |
|  | Lobería/Puerto | 62 | 30 | 60 | 95 | 61 | 9.02 | 1.37 |
|  | *Sum Floreana* | *790* | *553* | *597* | *708* | *729* |  |  |
| Española (8.28%) | Punta Suarez | 208 | 188 | 168 | 153 | 224 | 51.51 | 4.23 |
|  | Bahía Gardner | 226 | 54 | 116 | 106 | 111 | 31.80 | 2.67 |
|  | Punta Cevallos | 70 | 63 | 46 | 46 | 83 | 16.68 | 1.38 |
|  | *Sum Española* | *504* | *305* | *330* | *305* | *418* |  |  |
| San Cristóbal (30.80%) | Punta Pitt | 499 | 403 | 377 | 246 | 340 | 27.16 | 8.40 |
|  | Cerro Brujo | 85 | 85 | 90 | 90 | 89 | 6.46 | 1.99 |
|  | Isla Lobos | 156 | 186 | 219 | 269 | 211 | 15.40 | 4.72 |
|  | El Malecón | 629 | 637 | 611 | 616 | 616 | 45.67 | 14.07 |
|  | La Lobería | 87 | 52 | 78 | 63 | 83 | 5.31 | 1.61 |
|  | *Sum San Cristóbal* | *1456* | *1363* | *1375* | *1284* | *1339* |  |  |

The average percentage of each island population with respect to the archipelago is shown in parentheses. The average percentage of the population represented by each rookery relative to the island and archipelago are also shown. 2014-2018 DPNG database.

**Table S2.** Total counts (2014–2018) of Galapagos fur seal in breeding rookeries throughout the archipelago.

| **Island** | **Rookery** | **2014** | **2015** | **2016** | **2017** | **2018** | **% Rookery on island** | **% Rookery on archipelago** |
| --- | --- | --- | --- | --- | --- | --- | --- | --- |
| *Western region* |  |  |  |  |  |  |  |  |
| Fernandina (56.59%) | Cabo Hammond | 1204 | 548 | 836 | 751 | 1135 | 66.14 | 37.46 |
|  | Punta Douglas | 542 | 334 | 442 | 424 | 480 | 33.86 | 19.13 |
|  | *Sum Fernandina (FER)* | *1746* | *882* | *1278* | *1175* | *1615* |  |  |
| Isabela (21.31%) | Pta. Vicente Roca | 47 | 66 | 63 | 34 | 351 | 18.55 | 4.29 |
|  | Pta. Flores | 146 | 100 | 145 | 118 | 186 | 28.17 | 5.92 |
|  | Pta. Albermale | 92 | 39 | 99 | 35 | 87 | 13.88 | 2.94 |
|  | Cabo Marshall | 298 | 144 | 131 | 186 | 206 | 39.39 | 8.17 |
|  | *Sum Isabela (ISA)* | *583* | *349* | *438* | *373* | *830* |  |  |
| *Northern region* |  |  |  |  |  |  |  |  |
| Pinta (13.58%) | Cabo Chalmers | 363 | 298 | 247 | 299 | 357 | 100 | 13.58 |
| Marchena (3.03%) | Punta Calle | 26 | 116 | 32 | 32 | 140 | 100 | 3.03 |
| Genovesa (1.39%) | Bahía Darwin | 11 | 37 | 27 | 38 | 39 | 100 | 1.39 |
| *Central region* |  |  |  |  |  |  |  |  |
| Santiago (2.58%) | Puerto Egas | 91 | 41 | 62 | 54 | 53 | 100 | 2.58 |
| Rábida (1.54%) | Playa Roja | 23 | 37 | 34 | 25 | 59 | 100 | 1.54 |

The average percentage of each island population with respect to the archipelago is shown in parentheses. The average percentage of the population represented by each rookery relative to the island and archipelago are also shown. 2014-2018 DPNG database.

**Table S3.** Galapagos fur seal population sizes in breeding rookeries of the archipelago. Estimated values are based on corrected census data using correction factors for different age categories calculated for the GSL. The 95% confidence interval of each estimate is shown in parentheses.

| **Region/ Island** | **2014** | **2015** | **2016** | **2017** | **2018** | **% Island on Archipelago** |
| --- | --- | --- | --- | --- | --- | --- |
| **Western** 76.7% |  |  |  |  |  |  |
| Fernandina | 7303 | 3845 | 5165 | 4536 | 6584 | 54.6 |
|  | (6419-9120) | (3346-4880) | (4520-6502) | (3998-5642) | (5819-8147) |  |
| Isabela | 2446 | 1562 | 1847 | 1584 | 3603 | 22.1 |
|  | (2163-3021) | (1361-1979) | (1621-2311) | (1396-1969) | (3154-4528) |  |
| Sum Western Region | 9749 | 5407 | 7012 | 6119 | 10187 |  |
|  | (8582-12141) | (4707-6858) | (6140-8813) | (5393-7611) | (8973-12674) |  |
| **Northern** 10.1% |  |  |  |  |  |  |
| Pinta | 1681 | 1502 | 1007 | 1350 | 1716 | 14.5 |
|  | (1469-2117) | (1296-1929) | (883-1263) | (1183-1695) | (1489-2185) |  |
| Marchena | 94 | 613 | 115 | 122 | 652 | 3.2 |
|  | (85-112) | (529-789) | (104-135) | (110-144) | (570-819) |  |
| Genovesa | 55 | 168 | 125 | 159 | 166 | 1.3 |
|  | (48-70) | (148-209) | (110-155) | (143-193) | (148-201) |  |
| Sum Northern Region | 1830 | 2283 | 1246 | 1632 | 2533 |  |
|  | (1602-2299) | (1973-2927) | (1097-1553) | (1436-2032) | (2208-3205) |  |
| **Central** 4.2% |  |  |  |  |  |  |
| Santiago | 474 | 181 | 250 | 216 | 255 | 2.7 |
|  | (407-615) | (158-228) | (222-309) | (191-265) | (219-331) |  |
| Rábida | 96 | 174 | 150 | 99 | 243 | 1.5 |
|  | (85-118) | (152-217) | (132-184) | (89-117) | (215-302) |  |
| Sum Central Region | 570 | 354 | 400 | 314 | 498 |  |
|  | (492-733) | (310-444) | (354-493) | (280-382) | (434-632) |  |

**Figure S1**. Galapagos sea lion populations counted per region of the Galapagos Archipelago from 2014 to 2018. Dashed lines show the population trends in each region.


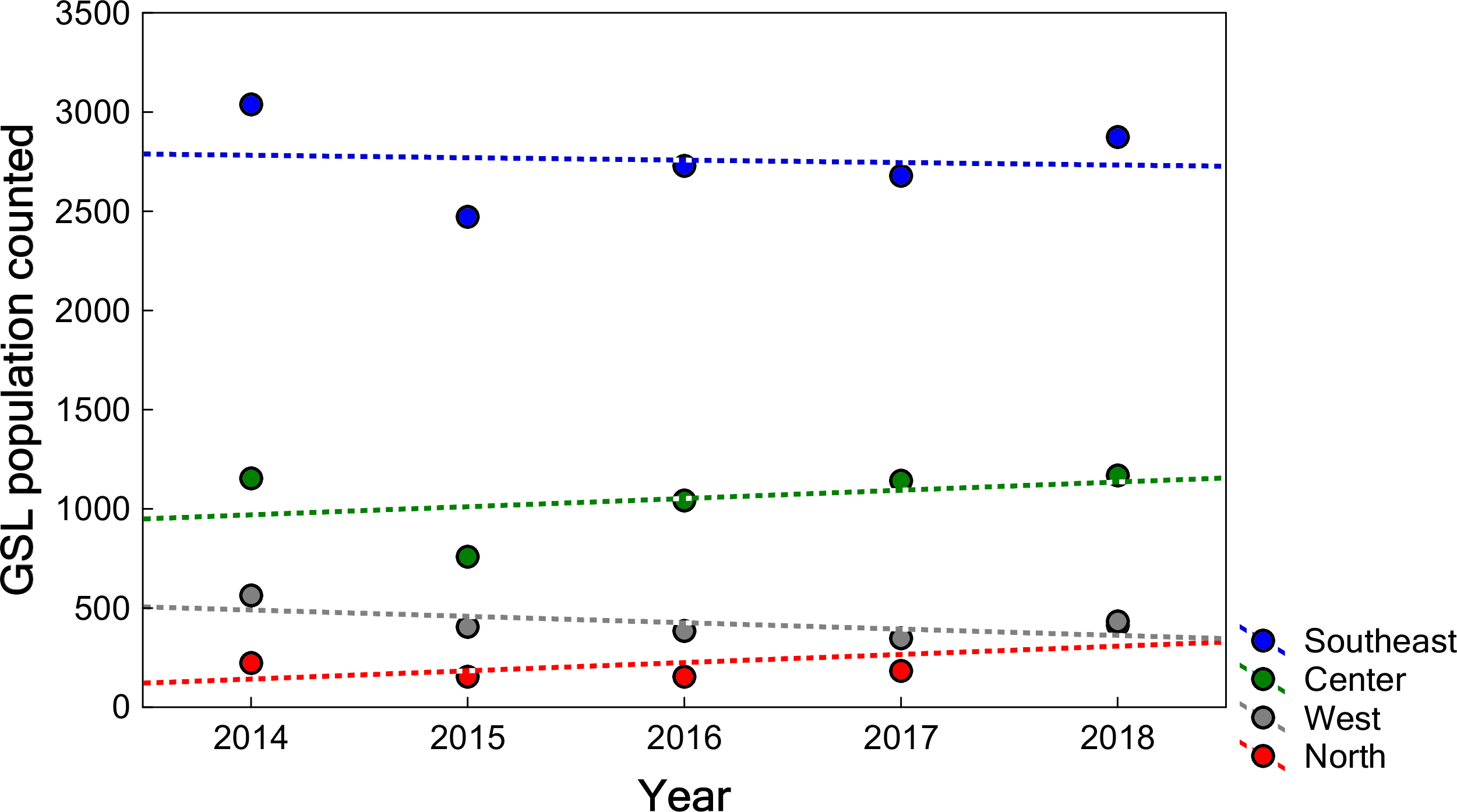


**Figure S2.** Galapagos fur seal populations counted per region of the Galapagos Archipelago from 2014 to 2018. Dashed lines show the population trends in each region which this species inhabits.


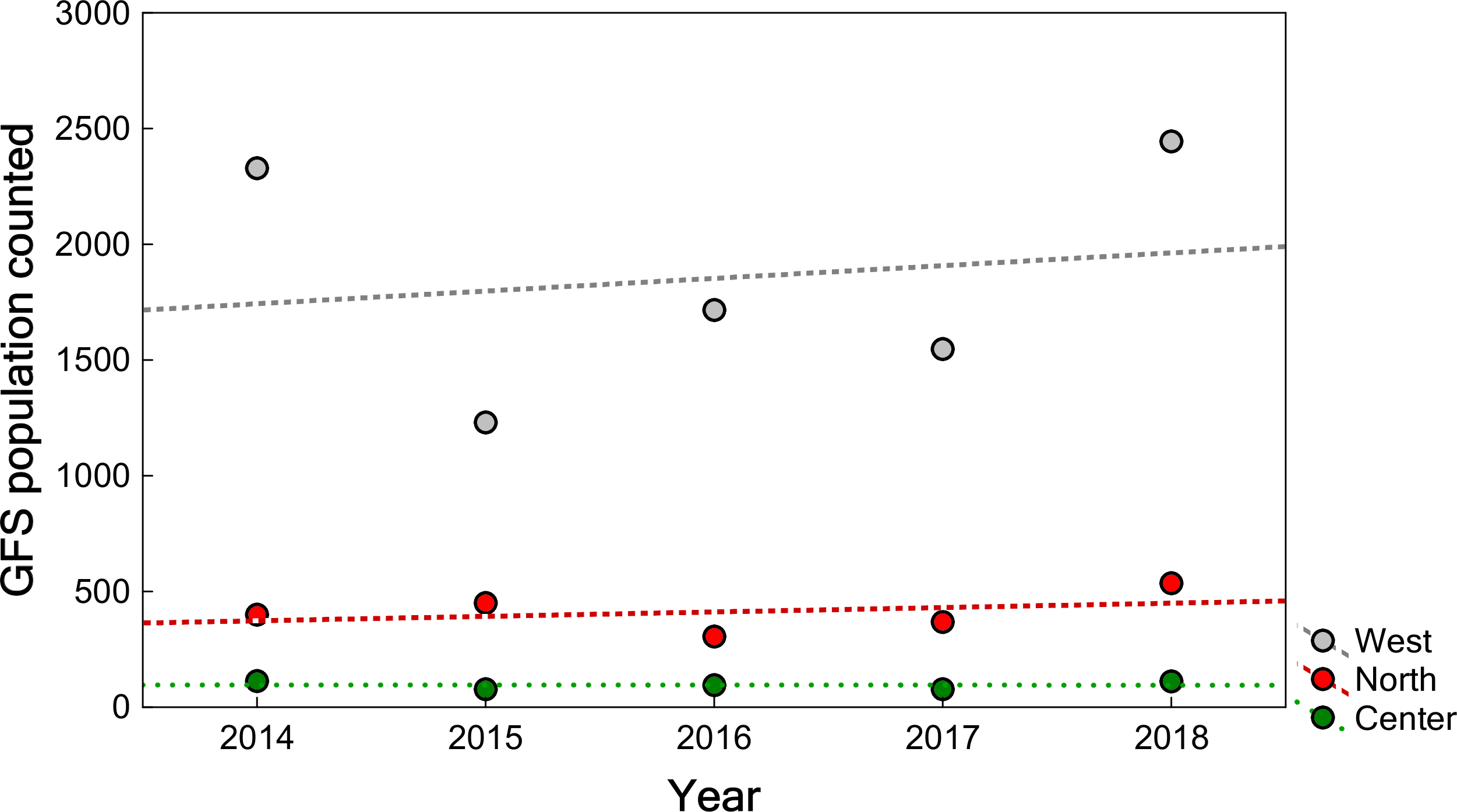

Supplement: Supplementary file 1 — Supplementary Information [file 41598_2021_88350_MOESM1_ESM.docx]
